# Synthesis, Crystal Structure, Quantum Chemical Analysis, Electrochemical Behavior, and Antibacterial and Photocatalytic Activity of Co Complex with Pyridoxal-(S-Methyl)-isothiosemicarbazone Ligand

**DOI:** 10.3390/molecules27154809

**Published:** 2022-07-27

**Authors:** Violeta Jevtović, Haneen Hamoud, Salma Al-Zahrani, Khalaf Alenezi, Salman Latif, Tahani Alanazi, Fahad Abdulaziz, Dušan Dimić

**Affiliations:** 1 Department of Chemistry, College of Science, University of Ha’il, Ha’il 81451, Saudi Arabia; v.jevtovic@uoh.edu.sa (V.J.); noonh.66@hotmail.com (H.H.); s.alzahrane@uoh.edu.sa (S.A.-Z.); k.alenezi@uoh.edu.sa (K.A.); sl.alanazi@uoh.edu.sa (S.L.); ty.alanazi@uoh.edu.sa (T.A.); fah.alanazi@uoh.edu.sa (F.A.); 2 Faculty of Physical Chemistry, University of Belgrade, Studentski trg 12–16, 11000 Belgrade, Serbia

**Keywords:** DFT, Co and Zn complexes, crystal structure, catalytic activity, biological activity

## Abstract

New complex Co(III) with ligand Pyridoxal-S-methyl-isothiosemicarbazone, (PLITSC) was synthesized. X-ray analysis showed the bis-ligand octahedral structure of the cobalt complex [Co(PLITSC-H)_2_]BrNO_3_·CH_3_OH (compound **1**). The intermolecular interactions governing the crystal structure were described by the Hirsfeld surface analysis. The structure of compound **1** and the corresponding Zn complex (([Zn(PLTSC)(H_2_O)_2_]SO_4_·H_2_O)) were optimized at the B3LYP/6–31 + G (d,p)/LanL2DZ level of theory, and the applicability was assessed by comparison with the crystallographic structure. The natural bond orbital analysis was used for the discussion on the stability of formed compounds. The antibacterial activity of obtained complexes towards *S. aureus* and *E. coli* was determined, along with the effect of compound **1** on the formation of free radical species. Activity of compound **1** towards the removal of methylene blue was also investigated. The voltammograms of these compounds showed the reduction of metal ions, as well as the catalyzed reduction of CO_2_ in acidic media.

## 1. Introduction

Pyridoxal-carbazones present an important class of ligands containing N and S donor atoms [1]. Their synthesis is fairly simple and usually includes condensation of ketone or aldehyde with thiosemicarbazide [1]. These compounds can also be considered as Schiff bases formed by the previously described procedure. Based on the ability to coordinate metal ions these compounds can be classified as mono- and bis-thiosemicarbazones. The presence of multiple donor atoms allows different binding modes depending on the stability of isomers and/or protonation/deprotonation [2,3,4,5,6,7,8]. Already at the beginning, those ligand systems seemed promising in terms of the ability to coordinate with transition metals, as well as the practical application, with prominent biological and catalytic activity. The coordination chemistry of these ligands was enriched with the potential antibacterial, antimalarial, antitumor, and antiviral activities of these ligands and their metal complexes [2].

**Scheme 1:**
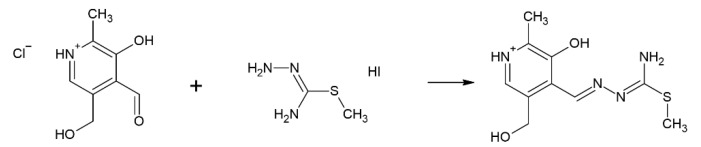
Synthesis of pyridoxal-S-methyl-isothiosemicarbazone (PLITSC).

**Scheme 2:**
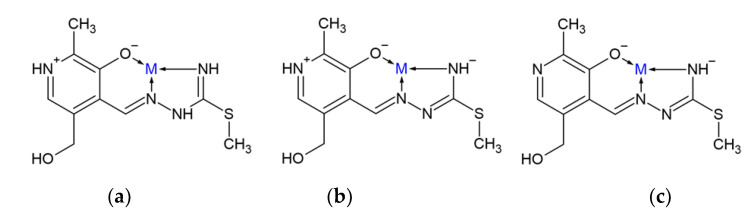
Coordination models and ligand forms: (**a**) neutral, (**b**) mono- and (**c**) dianionic.

The pyridoxal-S-methyl-iso-thiosemicarbazone (PLITSC) ligand is formed in the reaction between pyridoxal and S-methyl-isothiosemicarbazone (Scheme 1). The difference between thiosemicarbazones and isothiosemicarbazones is in the ability of a sulfur atoms to coordinate to transition metal ions, as proven in the crystallographic analysis [9]. Dehydration of PLITSC with pyridoxal moiety (3-hydroxy-5-hydroxymethyl-2-methyl-pyridine-4-carbaldehyde) results in the formation of Schiff base ligand PLITSC. Ligand PLITSC is a tridentate ligand, with the coordination sites being the oxygen of phenolic hydroxyl, hydrazine nitrogen, and amide nitrogen, creating the ONN form of the coordinated ligand. Coordinated PLITSC ligand is known to exist in three forms, namely neutral, mono- and dianionic (Scheme 2). The monoanionic form is obtained by deprotonation of hydrazine nitrogen, while the dianionic form is formed by the removal of pyridine hydrogen.

Numerous complexes formed between various metals and PLITSC are known in the literature. Several of them contain copper, such as ([Cu(NO_3_)(PLITSC)(H_2_O)](NO_3_) [10]), [Cu(PLITSC–2H)NH_3_]H_2_O·0.5MeOH [11] [Cu(ITSCPL-2H)(NH_3_)]_2_·2H_2_O [12], Cu(PLITSC)Br(CH_3_OH)]Br [13,14], Cu(PLITSC)Cl_2_, Cu(PLITSC)Br_2_ [8]. The coordination compounds with CuII are interesting because of the biological relevance of copper as an essential ion and antiviral, antitumor, and anti-inflammatory activity of these systems [15]. It has been shown that Cu complexes with pyridoxal semicarbazones and PLITSC can be used as a promising material for electrocatalytic hydrogen evolution [16]. The Cu complexes with morpholine–thiosemicarbazone hybrids specifically inhibited the growth of *S. aureus* with MIC value of 2–5 μg mL^−1^ and accumulated in the cancer cells through ribonucleotide reductase inhibition [17]. When Cu complexes with thiosemicarbazones are concerned, they are more efficient antitumor agents than cisplatin, with binuclear complexes being more potent than mononuclear [18]. The following Fe-containing complexes with PLITSC are described: Fe(PLITSC)Cl_3_·H_2_O, Fe(PLITSC)(PLITSCH)](NO_3_)_2_·H_2_O and [Fe(PLITSC)(PLITSC-H)]SO_4_·2H_2_O [19]. Cobalt complexes with PLITSC include the following: Co(PLITSC-2H)(PLITSC-H)]·CH_3_OH [20]; Co(PLITSC)Cl_2_]·2H_2_O, [Co(PLITSC)(PLITSC-H)]Cl_2_·4H_2_O and [Co(PLITSC-H)_2_]Cl·2H_2_O, [Co(PLITSC-H)(PLITSC-2H)]CH_3_OH, [Co(PLITSC)(NH_3_)_3_]NO_3_·3H_2_O [13]. Several compounds containing vanadium, molybdenum, and nickel were obtained, namely, [VO_2_(PLITSC–H)]·2H_2_O [21], [MoO_2_(PLITSC–2H)] [21], and [Ni (PLITSC) (H_2_O)_3_] (NO_3_)_2_ [22], respectively. Poladian and coworkers published a paper on the new Zn(II) complex with ligand derived from PLITSC in which the antioxidant activity of complex was higher than that of a Trolox [23]. The oxovanadium complex with similar ligands also showed better antioxidant activity than ascorbic acid [24]. Danac and coworkers also observed different binding modes of 2-formylpyridine S-methyl-isothiosemicarbazide depending on the present metal ion [25]. Similar coordination through the S atom was determined in the case of 2-acetylpyridine *N*^4^-allyl-*S*-methylisothiosemicarbazone, and its Cu complexes showed promising anticancer activity and high selectivity towards BxPC-3 cell lines [26]. The same group of researchers obtained several transition metal complexes with 2-formylpyridine *N*^4^-Allylthiosemicarbazone, of which Ni complex showed the highest selectivity towards Hl-60 cancer cell lines, and Zn complex had the highest selectivity towards RD cell lines [27]. The Zn and Cu complexes of the same ligand manifested high antibacterial activity [27]. In the last decade, several papers describing the good catalytic properties of the transition metal complexes with pyridoxal-carbazone [16,28,29,30,31,32,33] have been published.

This contribution presents results on the synthesis and structural characterization of a novel Co complex with pyridoxal-(S-methyl)-thiosemicarbazone ligand ([Co(PLITSC-H)_2_]BrNO_3_·CH_3_OH), analysis of intramolecular interactions governing the crystal stricture by Hirshfeld analysis, and stability interactions and optimization of a structure by quantum chemical methods. Special emphasis is put on the comparison with the Zn complex with the same ligand that is already published. The antibacterial activity of both compounds was determined and discussed. The kinetics of methylene blue removal by Co(PLITSC-H)_2_]BrNO_3_·CH_3_OH was also investigated, along with the effect of this compound on the formation of reactive oxygen species. The electrochemical behavior of both compounds is also analyzed. 

## 2. Results

### 2.1. Crystal Structure

**Scheme 3:**
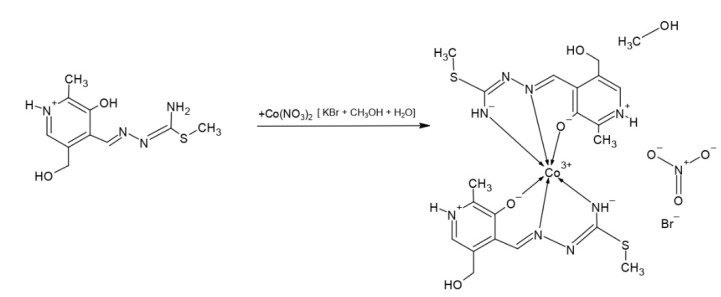
Synthesis of [Co(PLITSC-H)_2_]BrNO_3_·CH_3_OH.

**Figure 1:**
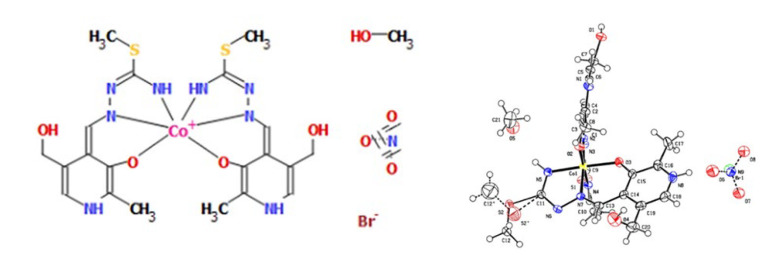
Octahedral geometry of Cobalt complex (**left**) and molecular structure of the complex compound **1** with the atom and ring-labeling scheme (**right**).

The cobalt complex [Co(PLITSC-H)_2_]BrNO_3_·CH_3_OH was obtained as explained in the Methodology section, according to the Scheme 3:

The cobalt complex [Co(PLITSC-H)_2_]BrNO_3_·CH_3_OH is a bis-ligand complex of octahedral geometry (Figure 1), with other crystallographic details presented in Table 1. Coordinated ligands are in the monoanionic form HL^−^. There is a methanol molecule in the outer sphere, along with the anions (bromide and nitrate). Considering the formula of the complex there are two ligands in the monoanionic form and the anions in the outer sphere, NO_3_^−^ and Br^−^ (0.45 and 0.55, respectively), with Co^3+^ as the central metal. The bond lengths and angles of the most important bonds including donor atoms of oxygen and nitrogen are shown in Table 2. It should be noted that complexes formed through a donation from S atoms could be possible, although they were not observed in this study. The slightly distorted octahedral geometry was obtained as expected, due to the presence of various donor atoms. 

As already mentioned, the synthesis was performed in a water–methanol mixture, which resulted in the appearance of methanol molecules in the outer sphere of the complex. Of particular interest is the formation in the outer sphere composed of nitrate and bromide anions, as well as their molar fraction in the gross formula. A newly obtained structure of cobalt complex with pyridoxal-S-methyl-isothiosemicarbazone is very similar to the structure already published earlier in 2011 [20]. The structure Co(PLITSC-2H)(PLITSC-H)]·CH_3_OH [20] also represents a bis-ligand complex, just two ligands in mono- and dianionic form are present. Additionally, anions NO_3_^−^ and Br^−^ are not present, because CoCl_2_ dissolved in methanol was used.

**Figure 2:**
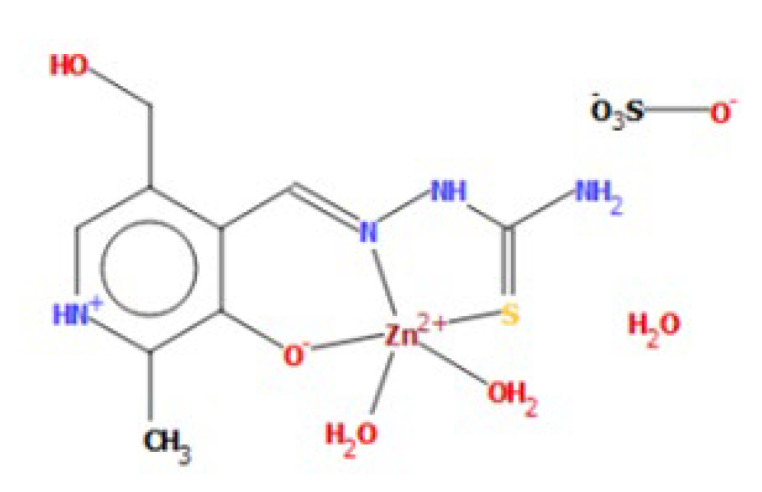
Square-pyramid geometry of Zinc complex (compound **2**) [14].

The structure of the zinc complex ([Zn(PLTSC)(H_2_O)_2_]SO_4_·H_2_O) (compound **2**) with the same ligand is described earlier [14]. The structural formula is presented in Figure 2. This complex has a square-pyramidal geometry with two water molecules, and coordination through S, N, and O atoms of ligand. It is important to observe that due to the structural features, the donation occurs from S atoms as well. This complex is included in this study, as the same type of ligand is used, and the effect of the metal ion on structural features and antibacterial activity can be discussed. 

### 2.2. IR spectra of Complexes

The IR spectrum of complex [Co(PLITSC-H)_2_]BrNO_3_·CH_3_OH, shown in Figure S1, confirms the previously described structure, in which Co^3+^ is surrounded by two PLITSC ligand molecules that are in the monoanionic form (HL), forming an octahedral geometry. In the outer sphere, there are half anions (NO_3_^−^ and Br^−^) which, considering their common share, contribute to the charge of −1 together. In the IR spectrum, there are two peaks located at 2751.70 cm^−1^ and 3112.30 cm^−1^ corresponding to the stretching NH^+^ vibration [34,35,36]. The absence of the characteristic peak for the neutral, zwitterionic form (H_2_L) of the ligand, at 2850 cm^−1^, also shows deprotonation of the ligand [37,38]. There is an intense band at 1372.10 cm^−1^, which is evidence of the presence of a nitrate group [39], as well as a peak in the range below 900, at approximately 650 [39], which corresponds to the vibrations of halogens, or in this case bromide. The peak at 3112.30 cm^−1^ corresponds to the O-H stretching vibration which belongs to the methanol molecule that is bound in the outer sphere of the complex [40,41].

The Zn^2+^ central metal is in a square-pyramidal geometry in coordination with the tridentate ligand PLTSC and two water molecules. The outer sphere consists of a sulfate group and uncoordinated water. The zinc complex is a mono-ligand complex, with the PLTSC ligand in a neutral form. Confirmation of the presence of the neutral form of the PLTSC ligand in coordination is the presence of a characteristic band at 2881 cm^−1^ for pyridoxal-semi-thiosemicarbazones [34,37], shown in Figure S2. The absence of a medium intense band in the spectrum at about 620 cm^−1^ explains the coordination of the SO_4_^2−^ group [39] and proves that this group is not coordinated to the central metal ion. Based on the absence of the ν (SH) band (about 2550 cm^−1^) [38,42] in the IR spectrum of the Zn complex, it can be concluded that the thiosemicarbazone complex also has a dominant thio-keto form in the solid state, which is in accordance with other thiosemicarbazone families of compounds. A tangle of vibrations can be attributed to vibrations of the heterocyclic ring, as well as δ(NH2) (probably about 1666 cm^−1^) and ν(C = N) chain (about 1505.48 cm^−1^) [35,43,44]. Finally, a clearly visible peak at 840 corresponds to coordinated water.

### 2.3. Hirshfeld Surface Analysis

**Figure 3:**
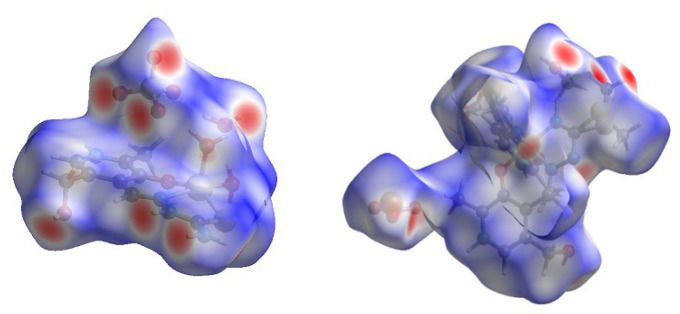
The Hirshfeld surfaces of compounds **2** (**left**) and **1** (**right**).

The crystallographic structure of compound **2** is almost exclusively stabilized by the interactions between atoms in the ligand (Figure 3 and Figure S3), as shown in the Hirshfeld surface analysis. There are no intermolecular interactions including the Zn ion, which proves that this ion is only surrounded by donor atoms of ligand. The most important interactions between ligands include hydrogen bonds between electronegative atoms (O, N, and S) and hydrogen. These contacts, H∙∙∙O, H∙∙∙N, and H∙∙∙S, account for 46, 5.1, and 2%, respectively (Figure S3).

This result is expected due to the presence of electronegative groups that include hydroxyl, carboxyl, amino functional groups, and sulfur atoms. Additionally, these interactions are formed with the water molecules that are in the first sphere of complex but are important for the bridging between layers. The interactions between H and C atoms are present at 6.5%. Different interactions can be described by this contact, for example, the interaction between a positively charged hydrogen atom and aromatic rings or carbon atoms bonded to the electronegative substituents. The rest of the contacts do not influence the structure significantly. 

Similar was observed for the stabilization interactions within compound **1** (Figure 3 and Figure S4), as the metal ion is well protected by the present ligands, and therefore, there were no interactions including the Co ion.

The number of hydrogen bonds formed between monomeric structures is even greater than for compound **2** as two PLITSC ligands coordinate to the same ion. The most frequent are weak H∙∙∙H interactions, which amount to 42.7%. The interactions between electronegative atoms of O, S, and N and H contribute significantly, namely 27.5, 5.8, and 9.4%, respectively. These interactions include hydrogen bonds between OH and amino groups, as well as sulfur atom interaction with OH groups of surrounding ligands. Weak interactions between carbon atoms and other elements amount to 5.8%, mostly weak hydrogen bonds H∙∙∙C (4.5%). The plethora of electronegative groups in both of these compounds leads to increased stability and potential biological application, as well as photocatalytic activity.

### 2.4. Structure Optimization

As mentioned in the Methodology section, the structures of complexes were optimized at the B3LYP/6-31G + (d,p) level of theory for H, C, N, and O atoms and the B3LYP/LanL2DZ for Co and Zn. The crystallographic structures were taken as starting ones and geometry optimization was performed without any geometrical constraints. The applicability of the given level of theory was investigated by comparing the experimental and theoretical bond lengths and angles. The parameters for comparison were the Mean Absolute Error (MAE) and Correlation Coefficient (R) [45,46]. Compound **2** is much simpler than the first one with a lower number of interactions. The R and MAE values for bond lengths are 0.998 and 0.02 Å, while for bond angles they are 0.81 and 4.4°, respectively (Tables S1 and S2). 

These results prove that the experimental parameters were well reproduced and that the selected level of theory is suitable for the description of the system. The experimental bond distance between Zn and two oxygen atoms of water molecules are 2.02 and 2.07 Å, while theoretical values are higher by only 0.1 Å. The distance between Zn and O, S, and N atoms of ligand are 1.94, 2.32, and 2.19 Å, respectively. These bond lengths are also 0.1 Å higher in the theoretical structure. These bonds are selected as the ligand itself is a very rigid structure with extended delocalization and a significant discrepancy between theory and experiment is not expected. On the other hand, bond angles changed to some extent due to the relaxation of the system and the absence of other compounds that are present in the experimental structure. These changes led to a somewhat lower value of the correlation coefficient, although the MAE value is low compared to the actual bond angles’ values. The most notable discrepancy was observed for the angles including oxygen atoms of water molecules, which is expected due to the flexibility and low mass of these molecules. For example, in the experimental structure, the angle between the oxygen atom of a water molecule, Zn, and sulfur/nitrogen donor atoms were 96.3 and 104.3°, while in the optimized structure, these values were 92.6 and 95.6°. During the optimization process, these angles are almost equilibrated. The rest of the angles including atoms of ligand, as well as within ligand, are negligibly different from the crystallographic ones. 

The structure of the Co complex is much more rigid with the symmetric position of ligands. The R and MAE values for the bond lengths/bond angles are 0.997 and 0.02 Å/0.993 and 1.24°, respectively (Tables S3 and S4). 

Again, the theoretical structure greatly resembles the crystallographic one. The experimental Co-O bond distances are 1.90 and 1.91 Å in the experimental structure, while in the theoretical structure they are 1.93 and 1.92 Å. The bond distances with nitrogen atoms of ligand are within 0.1 Å of the experimental values. The equilibration of structure is present to a certain extent after the optimization. When these results are compared, it should be borne in mind that the optimization is performed for an isolated structure in a vacuum and a crystal structure includes the surrounding molecules which influence the structure. The bond angles are also well reproduced with the R and MAE values being 0.993 and 1.24°. The most interesting are again the angles between the donor atoms. In this case, the differences between experimental and theoretical bond angles are between 1 and 2°, due to the overall stability and rigidity of the complex. The structure of the ligand is stabilized by a multitude of intramolecular and stabilization interactions, as explained in the following section; therefore, changes in bond lengths and angles are not expected, which is proved in this analysis. 

### 2.5. Stabilization Interactions and NBO Analysis

The stability of the complex depends greatly on the stability of the ligands involved. In the first section of NBO analysis special emphasis is put on the stabilization interactions within ligand, as electronegative atoms and elongated delocalization greatly influence the stability of ligand. Within the aromatic ring, the highest stabilization energies were calculated for π (C-N)→π* (C-C) and π (C-C)→π* (C-C) of around 70 kJ mol^−1^. The oxygen atom directly attached to the aromatic ring stabilizes the structure for 10.3 kJ mol^−1^. Strong delocalization between lone pair on the nitrogen atom and π (C-N) of the aliphatic chain is among the strongest, around 220 kJ mol^−1^, which is expected due to the presence of electronegative atoms and double bonds. The lone pair sulfur orbitals interact with π (C-N) with an energy of 120 kJ mol^−1^ leading to the delocalization in the parts that are distant from the aromatic ring. 

The coordination within compounds **1** and **2** occurs through lone pairs of oxygen and nitrogen atoms of ligand. The strongest interaction was obtained between the carbonyl oxygen and Zn ion with stabilization energy of 217 kJ mol^−1^. A nitrogen atom of the aliphatic chain contributes to the overall stability for 100 kJ mol^−1^. Much weaker interactions are formed between water molecules and Zn ions through lone pairs of oxygen. These interactions are on average 146 kJ mol^−1^. Similar was calculated for compound **1**, although water molecules were not present. This leads to the conclusion that compound **1** is much more stable as all of the interactions are formed between PLITSC and the central metal ion.

### 2.6. Photocatalytic Activity

**Figure 4:**
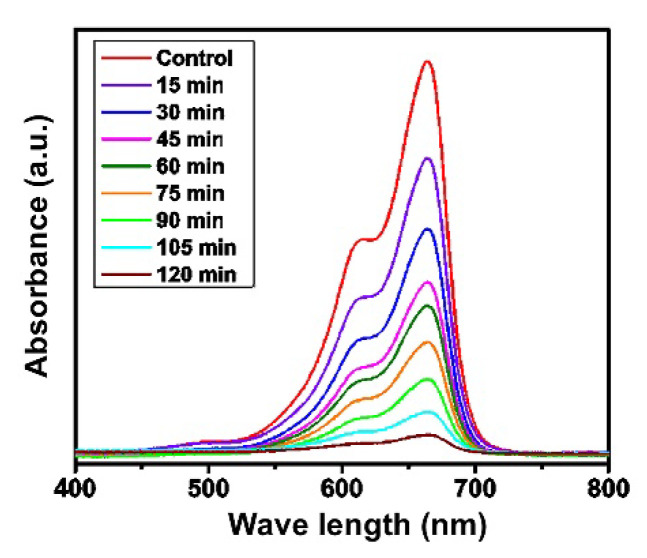
UV–visible analysis for the photodegradation of MB by compound **1**.

Pollutants are substances that create a negative impact on human society. Chemically, pollutants are of several types but industrial wastes mainly contain two prominent organic dyes, methylene blue (MB) and methyl orange (MO) [47,48,49]. In the human body, physiologic disorders along with several types of chronic diseases are mainly due to the MB released from industrial wastes [50,51]. In synthetic chemistry, due to the emergence of environmental issues, there is a focus on the synthesis of different catalysts that can be used for the removal of organic pollutants. The activity of compound **1** towards MB was analyzed in this paper. The removal of MB was followed by UV spectrophotometry after the irradiation of the reaction mixture with the UV light with characteristic absorption peaks at 465 nm and 414 nm. The results are shown in Figure 4 as the decrease in absorbance of MB in presence of compound **1** within 120 min from the reaction start. Based on the absorbance values, it can be concluded that up to 90% of dye was removed within the given time, which classifies compound **1** as a very potent catalyst. The absorbance at 665 nm was used to construct the kinetic curve for the preliminary determination of the removal kinetics [52]. The results shown in Figure S5 verify that the removal of MB follows the first order kinetics, with a reaction rate of 3.6 × 10^−4^ s^−1^. It can be assumed that this is pseudo-first-order kinetics that is dependent on the amount of added compound **1**. The determined reaction order kinetics was also observed in similar research [52,53]. 

The enhanced activity of compound **1** can be multifactorial. It can be discussed that the formation of free radical species due to the catalytic reaction on the surface of compound **1** can be one of the possible reaction mechanisms. To verify the assumption of the reactive oxygen species formation, the measurements explained in Section 2.9. were performed. Moreover, the high surface area and the intensity of the light are also important factors in increasing the catalytic efficiency of the synthesized catalyst [54]. Additional theoretical research is needed for the possible understanding of the removal mechanism.

### 2.7. Antibacterial Activity

New therapies with potent compounds are needed for various microorganisms as their resistivity increases with the overuse of the available medicine [55,56]. For this purpose, the antibacterial activity of compounds **1** and **2** was tested against Gram-negative and Gram-positive bacteria. The observed zone of inhibitions for *E. coli* and S. *aureus* bacterial strains for compounds **1**, **2,** and Streptomycin are shown in Table 3.

The activity of the title complex towards Gram-positive and Gram-negative bacteria is evident from the presented results. There is an increase in activity with an increase in the concentration of compound **1**. The zone of inhibition of *S. aureus* increases from 7 to 17 mm for the concentration increase from 0.25 to 2 mg mL^−1^, while in the case of *E. coli*, these values range from 5 to 14 mm. Partially, the activity can be a consequence of the methanol presence in the structure, although this is less probable. Lower activity was observed for compound **2**. The zones of inhibition in the case of *S. aureus* and *E. coli* are between 0.3 and 4.8 mm and 0.2 and 3.4 mm, respectively. For the positive control, Streptomycin, the zone of inhibition was 15 mm for the concentration of 0.25 mg mL^−1^ in the case of *S. aureus* and 13 mm in the case of *E. coli*. Therefore, compound **1** can be considered a moderately active compound for both types of bacteria. On the other hand, the activity of compound **2** is low compared to both standard and compound **1**. The basic difference between Gram-negative and Gram-positive bacteria is the chemical composition of the cell wall. The cell wall of Gram-negative bacteria is chemically composed of thin peptidoglycan and lipopolysaccharide cell wall while the cell wall of Gram-positive bacteria is chemically composed of multilayers of peptidoglycan which, in turn, provided the thickness to the cell wall in comparison to Gram-negative bacteria. According to the previous literature survey, the thiosemicarbazones and their metal complexes are usually effective on *S. aureus*, *S. epidermidis*, *E. coli*, and other microbial strains [57,58,59], although a significant effect of metal ion was observed, which is consistent with the results of this study. As the results showed moderate activity of obtained compound towards the selected microbial stains, further biological studies are advised.

### 2.8. Minimum Inhibition Concentration (MIC)

The MIC of compound **1** was determined toward *E. coli, P. aeruginosa,* and *S. aureus* in triplicates using a serial dilution approach [60]. A gradient concentration sequence of compound **1** (20 to 60 µg/mL) was taken. The MIC against *E. coli and P. aeruginosa* was found 30 µg mL^−1^ while against *S. aureus* was 20 µg mL^−1^ as shown in Table 4. The MIC values of compound **2** for the same bacterial strains were taken from reference [14]. As it can be seen, the MIC values for compound **2** were much higher, which is in accordance with previous experiments. The MIC values for the positive control, chloramphenicol, are lower than for compound **1** [14], which again proves the moderate activity of compound **1** and low activity of compound **2** towards selected microorganisms. 

### 2.9. Measurement of Reactive Oxygen Species

**Figure 5:**
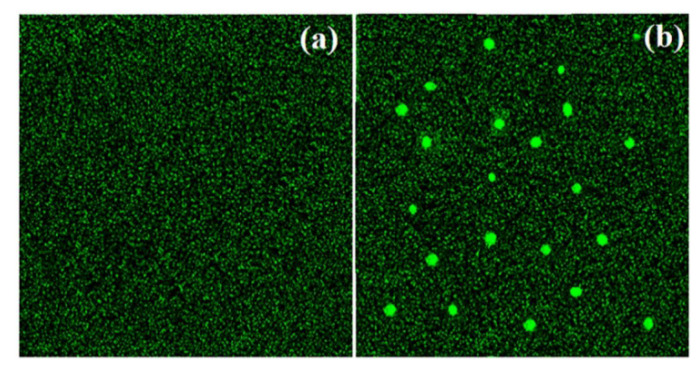
Examination of ROS in the (**a**) absence and (**b**) presence of compound **1**.

The antibacterial activity of compound **1** could be a consequence of the reactive organic species generation. Reactive organic species in the form of superoxide ion (O_2_^−^), hydroxyl radical (OH), and hydrogen peroxide (H_2_O_2_), could be produced in the bacterial cell by the excitation of electrons in compound **1**, which ultimately leads to the destruction of cell components, mainly proteins, DNA and cell membrane. To verify this assumption, the experiments with 2, 7-dichlorofluorescein-diacetate were performed. During the process, reactive oxygen species cause oxidation of 2, 7-dichlorofluorescein-diacetate into dichlorofluorescein. The process was clarified by the appearance of green fluorescence upon excitation at 488 nm [61], Figure 5. The interesting features depicted in Figure 5b show the intensified green fluorescence upon treatment of the sample with compound **1**, which is the due to the formation of the reactive species. These reactive organic species destroy the intracellular system of the cell, especially DNA, cell membrane, and the enzymatic system, which ultimately leads to the death of the cell.

### 2.10. Cyclic Voltammetry of [Co(PLITSC-H)_2_]BrNO_3_ •CH_3_OH

**Figure 6:**
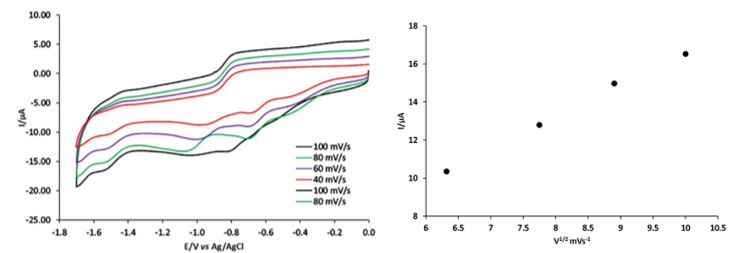
Cyclic voltammograms of 0.45 mM [Co(PLITSC-H)_2_]BrNO_3_ CH_3_OH at carbon electrode, at different scan rates in DMF containing 0.2 M [NBu_4_][BF_4_] and ips versus the square-root of the scan rate.

Cyclic voltammetry of compound **1** under nitrogen at carbon electrode (Figure 6) exhibited three waves at −1.5, −1.1 and −0.6 V vs. Ag/AgCl. The values for the potential are given in Table S5. These waves correlate with the formation of Co(III)/Co(II), Co(II)/Co(I) and Co(I)/Co(0), respectively. This electrochemical analysis was conducted on the third wave reduction of [Co(PLITSC-H)_2_]BrNO_3_·CH_3_OH. A diffusion-controlled reversible one-electron step comprises the third reduction reaction step. Thus, (i) the plot for the peak current I_ip,red_ vs. ν^1/2^ is linear (Figure 6, right), with an intercept close to zero, which indicates no chemical reaction and only an electron transfer. The diffusion coefficient for 0.45 mM of compound **1** is estimated to be 2.7 × 10^−3^cm^2^ s^−1^, according to Randles-Sevcik Equation [62]:

Ip = −(2.69 × 105) n^3/2^ C0 D^1/2^ v^½^ (1)

where C0 is the bulk concentration in mol/cm^3^, D is the diffusion coefficient in cm^2^ s^−1^, v is the potential scan rate in V s^−1^, and I is the current density.

**Figure 7:**
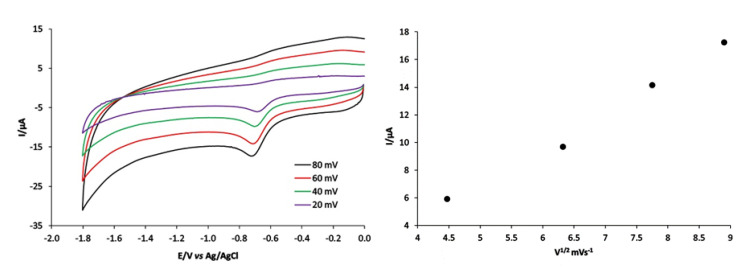
Cyclic voltammograms of 0.45 mM [Co(PLITSC-H)_2_]BrNO_3_ CH_3_OH at carbon electrode, at different scan rates in DMF containing 0.2M [NBu_4_][BF_4_] and ips versus the quare-root of the scan rate.

The electrochemical behavior of compound **2** was investigated under similar conditions (Figure 7). These voltammograms contain two waves at −1.65 and −0.7 V Ag/AgCl, corresponding to the formation of Zn (II)/Zn (I), Zn (I)/Zn (0), respectively. Additionally, the diffusion coefficient for 0.41 mM solution of compound **2** was estimated to be 2.4 × 10^−3^ cm^2^ s^−1^ from the plot presented in Figure 7, right.

### 2.11. CV of Compound **1** under CO_2_

**Figure 8:**
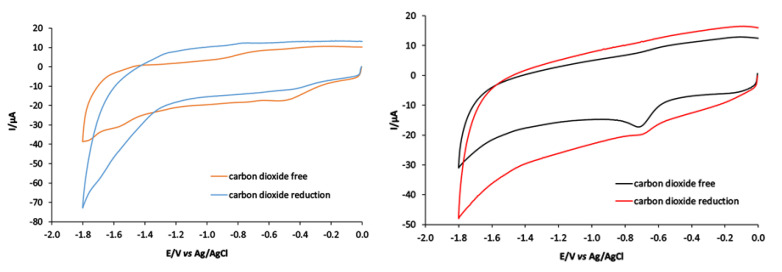
CV of 0.4 mM solution of compounds **1** and **2** at a vitreous carbon electrode in DMF 0.2 M [NBu_4_][BF_4_]; scan rate 100 mV s^−1^ under N_2_ and CO_2_ at 1 atmosphere.

The electrochemical behavior of compound **1** under CO_2_ was investigated. The third reduction peak of Co(III)/Co(II) (−1.55 V vs. Ag^+^/AgCl) was monitored in Figure 8. Notably, the peak of Co(III)/Co(II) can reduce CO_2_ into CO. The electrocatalytic conversion was carried out in vitreous carbon in the absence and presence of acid sources as represented in Figure 8, where CO_2_ interacted with the Co(III)/Co(II) wave of compound **1**. The third peak reduction of Co(III)/Co(II) increased in the presence of CO_2_. The peak current in the absence of CO_2_ was 5 µA, which increased to 25 µA upon the addition of CO_2_. Under the same condition, ([Zn(PLTSC)(H_2_O)_2_]SO_4_·H_2_O) was used as a catalyst for CO_2_ reduction. It was found that CO_2_ interacted with Zn(I)/Zn(0)) at −1.30 V vs. Ag^+^/AgCl, as shown in Figure 8.

### 2.12. Magnetic Moment Measurements and Molar Conductivity of Complexes

The magnetic moment measurements were performed for both compounds **1** and **2**. These resulted in magnetic moments of μeff = 0 BM for both complexes, which corresponded to a Co (III) d^6^ and Zn (II) d^10^ (diamagnetic) center. The molar conductivity value for Co complex (λ_M_ (H_2_O) = 195 Scm^2^ mol^−1^) was greater than for a 1:1 type of electrolyte, which was a consequence of the cation which is triply charged and anions in the solution. In the case of compound **2**, the molar conductivity was λ_M_ (H_2_O) = 160 Scm^2^ mol^−1^ which is lower than for compound **1**, as expected due to the present cations and anions. 

## 3. Materials and Methods

All commercially obtained reagent-grade chemicals were used without further purification, except for the ligands, which were prepared according to the previously described procedures [37].

### 3.1. Synthesis of [Co(PLITSC-H)_2_]BrNO_3_·CH_3_OH (1)

Compound **1** ([Co(PLITSC-H)_2_]BrNO_3_·CH_3_OH) was obtained under mild conditions. An amount of 0.01 mol of PLITSC ligand was dissolved in 15 cm^3^ of water, with heating, while 0.01 mol Co(NO_3_)_2_ was dissolved in 15 cm^3^ CH_3_OH and added to the first solution. Additionally, 0.005 mol KBr was added to the solution. A clear purple solution was left at room temperature to crystallize by employing the method of slow evaporation. After a few hours, purple crystals appeared; yield: 0.16 g (65%).

### 3.2. IR Spectroscopy, Magnetic Susceptibility, Molar Conductivity, and Elemental Analysis

IR spectra (KBr disk) were recorded on a Thermo Nicolet (NEXUS 670FT-IR) instrument, Department of Chemistry, University Hail. Using a magnetic susceptibility balance (Johnson Matthey Chemicals Limited, England), magnetic susceptibilities were measured at room temperature. Molar conductivities of the freshly prepared 1 × 10^−3^ M solution were measured on a Jenway 4010 conductivity meter. Elemental (C, H, N) analysis of air-dried samples was carried out by standard micro methods in the Centre for Instrumental Analysis, Faculty of Chemistry, Belgrade. 

Anal. Calcd. for: [Co(PLITSC-H)_2_]BrNO_3_·CH_3_OH (C21 H30 Br0.55 Co N8.45 O6.35 S2): C 37.68, H 4.51, N 17.68 Found: C 38.05, H 4.78, N17.95.

Anal. Calcd. for: [Zn(PLTSC)(H_2_O)_2_]SO_4_·H_2_O (C19 H18 N4 O9 S2 Zn): C 50.07, H 3.98, N 12.29. Found: C 49.63, H 4.00, N12.85.

### 3.3. X-ray Analysis

A single crystal (0.12 × 0.09 × 0.17) was examined at 296 K. For the X-ray measurements, a single crystal of the complex was mounted on glass fiber and examined at 296 K on a Bruker D8 Venture APEX diffractometer equipped with Photon 100 CCD area detector using graphite-monochromatic Mo-Kα radiation [λ = 0.71073 Å]. In general, in the difference map, the hydrogen atoms were 109 visible. Hydrogen atoms bound to carbon were initially positioned geometrically, while the hydrogen atoms for the coordinated water molecules were located in the difference map. All hydrogen positions and isotropic displacement parameters were then refined in a separate cycle. Hydrogen positions were checked for feasibility by examination of the hydrogen-bonding network. Crystallographic data in the Cambridge Crystallographic Data Centre (CCDC, 12 Union Road, Cambridge CB2 IEZ, UK; e-mail: depos-it@ccdc.cam.ac.uk) were deposited (CCDC number 2,164,543). Crystal data collection and structure refinement are given in Table 1.

### 3.4. Photocatalytic Activity

The photocatalytic effectiveness of the newly designed Co complex was examined in this contribution. For this purpose, compound **1** was designed and treated with methylene blue (MB) which is considered an organic pollutant. To carry out the activity, 5 g of compound **1** was introduced in a flask containing 80 mL of 10 ppm MB solution. Before irradiation, the mixture was whirled to achieve the best possible equilibrium between the complex and dye. In this experiment, a control solution was also prepared to contain only compound **1**. Both solutions were separately irradiated using a visible light source (300 W halogen lamp) to measure the breakdown of MB and the aliquots were taken at regular time intervals.

### 3.5. Antibacterial Activity

Agar well diffusion route was considered the most used and prominent technique utilized to determine the antibacterial activity of compounds **1** and **2 [63,64]**. In this method, sterile swabs were used to obtain the proper lining of the *Staphylococcus aureus* (*S. aureus)* and *Escherichia coli* (*E. coli*) inocula on Muller–Hinton plates. With the help of a cork borer, 6 mm wells were created in the agar nutrient plates. Then, solutions of different concentrations (i.e., 0.25, 0.5, 1, 2 mg/mL) of compound **1** were prepared and 50 µL of the solution was transferred into each well. In the whole process, Streptomycin was chosen as standard, and the plates were placed in an incubator under specialized conditions of 37 °C for 24 h.

### 3.6. Minimum Inhibition Concentration (MIC)

MIC value of compound **1** was calculated by using the serial dilution method [65]. In this method, the concentration of compound **1** is systematically reduced using a fixed volume of a liquid diluent. For this purpose, bacterial strains of *S. aureus* and *E. coli* strains of 2 mL were prepared in six different test tubes. After that, the test tubes were spilled with 2 mL of compound **1** solution. The chloramphenicol was used as a positive control. Finally, the six test tubes were incubated under optimal conditions at 37 °C. The solutions were left for 24 h, and all measurements were performed as triplicates. After incubation, the lowermost concentration where the growth of the corresponding microorganism was no longer noticeable with a Beckman DU-70 UV-Vis Spectrophotometer was taken as MIC.

### 3.7. Determination of ROS

The 2, 7-dichlorodihydrofluorescein diacetate (DCFH-DA) test is used for measuring the intracellular formation of ROS. To determine the oxidative stress effects of compound **1**, the fluorescent property of the mentioned due was utilized. In the experiment, the solution of varying concentrations of compound **1** was prepared with the E. coli strains and further kept in an incubator for 3 h at 300 rpm. Onwards, the suspension of the bacterial cells was collected under 1000 rpm for 15 min and subsequently washed with PBS solution. The pellet suspension was further mixed with 1 cm^3^ of 20 mM concentration of DCFH-DA. Finally, the whole mixture was washed with PBS to remove the unwanted coloring agents. The fluorescence spectrophotometry technique was implemented to study the fluorescence intensity based on two wavelengths (488 (excitation wavelength) and 535 (emission wavelength)).

### 3.8. Cyclic Voltammetry

Cyclic voltammetry experiments were carried out using an Autolab PGSTAT 128 potentiostat. The electrochemical cell containing 5 mL of a solution of electrolyte [NBu4][BF4], 0.2 M in DMF, was degassed with nitrogen gas. A conventional three-electrode arrangement was employed, consisting of a vitreous carbon working electrode (CPE) (0.07 cm^2^), a platinum wire as the auxiliary electrode, and Ag/AgCl as a reference electrode.

### 3.9. Theoretical Calculations

The geometry optimization of Zn and Co complexes was performed in the Gaussian program package [66]. The global hybrid Generalized Gradient Approximation (GAA) functional B3LYP was used in combination with the 6–31 + G(d,p) basis set for H, C, and N atoms and LanL2DZ basis set for Zn and Co. The structure optimization started from the crystallographic structure, and the calculations were performed at 298 K without any geometrical constraints. The absence of imaginary frequencies proved that the equilibrium geometry was found. The natural bond orbital analysis [67], as implemented in the Gaussian program package, was performed to investigate the stabilization interactions within a structure that lead to the overall stability, especially within ligand and between ligand and central metal ion. 

The interactions within crystal structure are important for the overall stabilization. Hirsfeld surface analysis is a method for their analysis through the contacts of interacting atoms. The crystal structure was investigated in the Crystal-Explorer [68]. Hirsfeld surface is represented by a graph showing two distances, one between two nearest nuclei (d_e_) and the second one being the distance from nuclei to the external surface (d_i_) [40,69]. The normalized distance (d_norm_) is then colored in red, white, and blue if the shown distance is shorter, equal, or longer than the Van der Waals separation between atoms, respectively. In this contribution, the d_norm_ is presented in the range between −0.4939 au (red) and 1.1572 au (blue). The fingerprint plots for selected atom pairs are given in the Supplementary Information only for the most abundant contributions. 

## 4. Conclusions

A bis-ligand–cobalt complex ([Co(PLITSC-H)_2_]BrNO_3_·CH_3_OH) (compound **1**) with octahedral geometry was synthesized. The coordinated ligands are in monoanionic form, while in the outer sphere nitrate and bromide ions are present. The structural features of this complex differ significantly from the Zn complex with the same ligand, [Zn(PLTSC) (H_2_O)_2_]SO_4_·H_2_O] (compound **2**), which has a square-pyramidal geometry. The IR spectra of compounds proved the presence of monoanionic forms of ligands. The Hirsfeld surface analysis of these compounds showed that hydrogen bonds were responsible for the stabilization of crystallographic structure through H∙∙∙O, H∙∙∙N, and H∙∙∙S contacts. The structures were optimized at B3LYP/6-31G + (d,p) for H, C, N, and O atoms and the B3LYP/LanL2DZ level of theory for Co and Zn. The comparison between experimental and theoretical bond lengths and angles proved the applicability of the chosen level of theory. High correlation factors and low mean absolute errors between the experimental and theoretical bond lengths and angles were used as quantitative parameters for comparison. Significant stabilization interactions were observed in the ligand structure, mostly through extended delocalization. The photocatalytic degradation of methylene blue in presence of compound 1 followed a first-order reaction with a reaction rate of 3.6 × 10^−4^ s^−1^ and within time intervals of 0 to 120 min, the concentration of dye was reduced by more than 90%. In the concentration range between 0.25 and 2 mg mL^−1,^ the inhibition zones of compound **1** were between 7 and 17 mm (*S. aureus*) and 5 and 14 mm (*E. coli*). For the same concentration range, compound **2** showed much lower inhibition zone values. The standard drug, Streptomycin, had an inhibition zone of 15 mm for the lowest concentration, which proves the moderate activity of compound **1**. The minimum inhibitory concentrations of compound **1** were comparable to another standard drug, Chloramphenicol, when activity towards *S. aureus*, *E. coli*, and *P. aeruginosa* is concerned. The antibacterial activity is probably due to the formation of radical species, as shown in the presence of 2, 7-dichlorofluorescein-diacetate. The cyclic voltammograms of the title compound had three peaks corresponding to Co(III)/Co(II), Co(II)/Co(I), and Co(I)/Co(0) reductions. On contrary, the reduction of Zn was observed through two peaks. The interactions with CO_2_ were much higher in the case of compound **1**.

## Figures and Tables

**Table 1:** Crystal data and structure refinement details of compound **1**.

Empirical Formula	C21 H30 Br0.55 Co N8.45 O6.35 S2
Formula weight	669.43
Temperature	123 K
Wavelength	0.71073
Crystal System	monoclinic
Space group	P-1
Volume	1386.27 (5)
Unit cell dimension	a = 8.7000 (2); b = 10.8488 (2); c = 15.9018 (3);
Z	Alpha = 74.505 (1); Beta = 82.126 (1); Gamma = 73.881 (1)

**Table 2:** The bonds length and Angles [°] of Co complex.

The Bonds Length [Å]	Angles [°]
Co1-O2 1.911 (2)	O2-Co1-O3 87.50 (6)
Co1-O3 1.879 (1)	O2-Co1-N2 95.56 (7)
Co1-N2 1.877 (1)	O2-Co1-N7 88.64 (7)
Co1-N7 1.882 (2)	O2-Co1-N4 177.58 (7)
Co1-N4 1.885 (2)	O2-Co1-N5 92.97 (7)
Co1-N5 1.887 (2)	O3-Co1-N2 88.31 (7)

**Table 3:** Zone of inhibition of compound **1** and standard drug against tested bacteria.

Microorganisms	Concentration (mg mL^−1^)	Zone of Inhibition (mm)			
		Compound 1	Compound 2	Streptomycin	Negative Control
*S. aureus*	0.25	7.0 ± 0.2	0.3 ± 0.2	15 ± 0.3	Zero inhibition
	0.5	11.0 ± 0.3	0.8 ± 0.3		
	1	14.0 ± 0.3	2.6 ± 0.3		
	2	17.0 ± 0.4	4.8 ± 0.4		
*E. coli*	0.25	5.0 ± 0.1	0.2 ± 0.1	13 ± 0.2	Zero inhibition
	0.5	8.0 ± 0.2	0.9 ± 0.3		
	1	11.0 ± 0.4	1.9 ± 0.3		
	2	14.0 ± 0.4	3.4 ± 0.4		

**Table 4:** MIC of Cobalt(III) pyridoxal-S-methylisothiosemicarbazone complex against tested bacteria for various concentrations, compound **2**, and chloramphenicol.

Bacteria	Tested Sample (μg mL^−1^)						
	60	50	40	30	20	Compound 2	Chloramphenicol
*S. aureus*	−	−	−	−	−	>1000	5
*E. coli*	−	−	−	−	+	>1000	5
*P. aeruginosa*	−	−	−	−	+	>1000	50

+ represents partial inhibition of bacteria); − represents strong inhibition of bacteria).

## Data Availability

Not applicable.

## Supplementary Materials

The following supporting information can be downloaded at: https://www.mdpi.com/article/10.3390/molecules27154809/s1, Figure S1: IR spectrum of [Co(PLITSC-H)_2_]BrNO_3_·CH_3_OH 2; Figure S2: IR spectrum of [Zn(PLTSC)(H_2_O)_2_]SO_4_·H_2_O. Figure S3: The fingerprint plots for the specific interactions within the Hirshfeld surface analysis of compound **2**. Figure S4: The fingerprint plots for the specific interactions within the Hirshfeld surface analysis of compound **1**. Figure S5: The kinetic curve for the reduction of methylene blue in presence of compound **1**. Table S1: Crystallographic and optimized bond lengths (Å) for compound **2**; Table S2: Crystallographic and optimized bond angles (°) for compound **2**; Table S3: Crystallographic and optimized bond lengths (Å) for compound **1**; Table S4: Crystallographic and optimized bond angles (°) for compound **1**; Table S5: Reduction wave potentials of [Co(PLITSC-H)_2_]BrNO_3_·CH_3_OH and ([Zn (PLTSC) (H_2_O)_2_]SO_4_·H_2_O) versus Ag|AgCl[NBu4][BF4]–DMF.

## Sample Availability

Samples of the title compounds are available from the authors.
