# Supplementary material for: Synthesis, Crystal Structure, Quantum Chemical Analysis, Electrochemical Behavior, and Antibacterial and Photocatalytic Activity of Co Complex with Pyridoxal-(S-Methyl)-isothiosemicarbazone Ligand
Source: Molecules. 2022 Jul 27;27(15):4809. doi: 10.3390/molecules27154809 (PMC9369583; doi:10.3390/molecules27154809)
Supplement: Supplementary file 1 [file molecules-27-04809-s001.zip › molecules-1836254-supplementary.pdf]

Supplementary information for:

Synthesis, crystal structure, quantum chemical analysis, electrochemical behavior, antibacterial and photocatalytic activity of Co complex with pyridoxal-(S-methyl)-isothiosemicarbazone ligand

Violeta Jevtovic[1], Haneen Hamoud[1], Salma Alzahrani[1], Khalaf Alenezi[1], Salman Latif[1], Dusan Dimic[2]

[1]Department of Chemistry, College of Science, University of Ha'il, Ha'il 81451, Kingdom of Saudi Arabia

[2] University of Belgrade – Faculty of Physical Chemistry, Studentski trg 12-16, 11000 Belgrade, Serbia

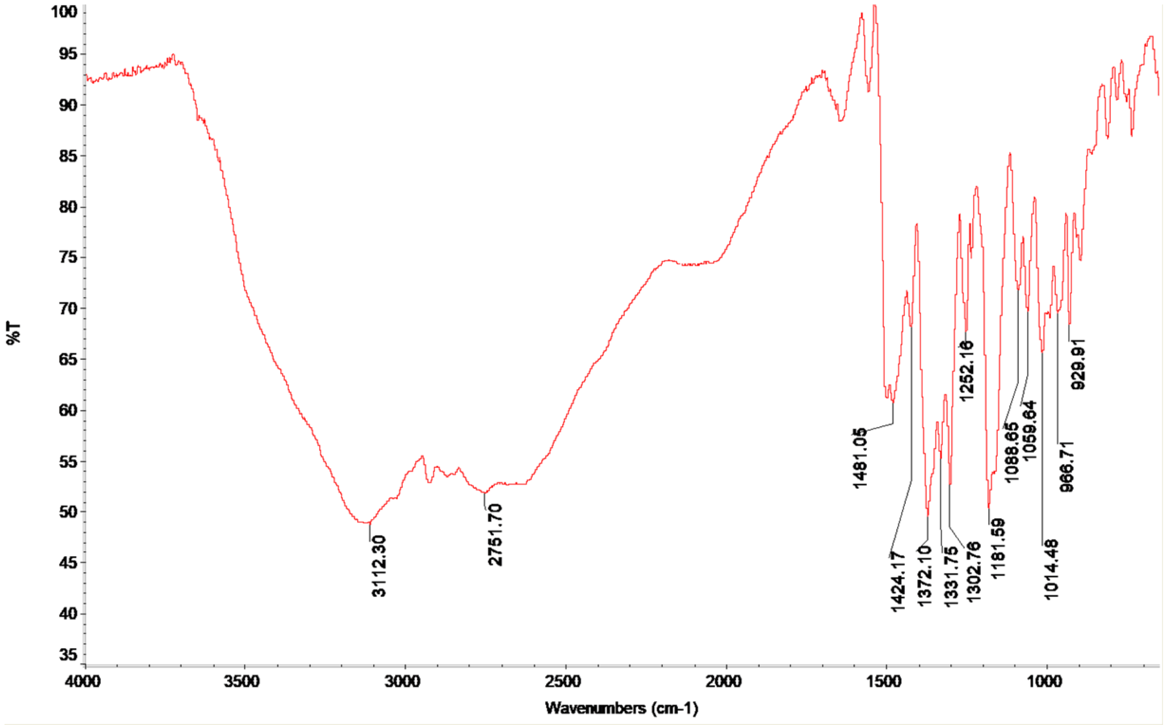

Figure S1. IR spectrum of **[Co(PLITSC-H)$_2$]BrNO$_3$·CH$_3$OH.**

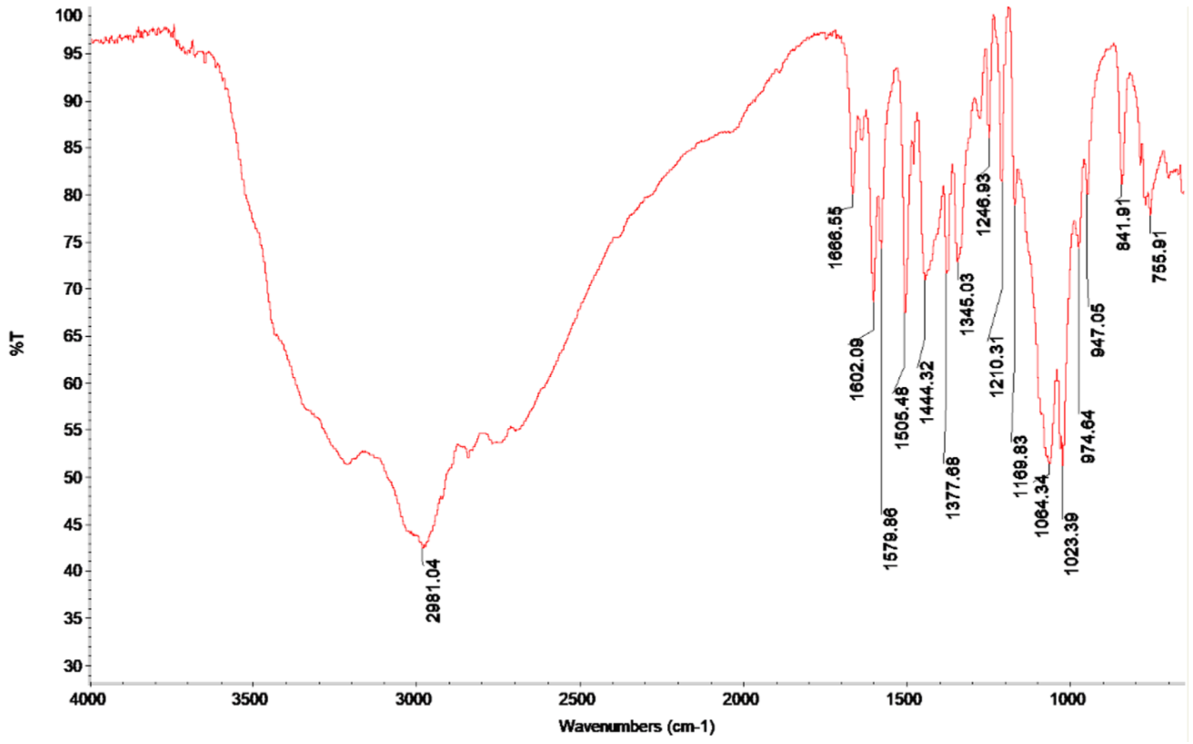

Figure S2. IR spectrum of **[Zn(PLTSC)(H_2O)$_2$]SO_4·H_2O.**

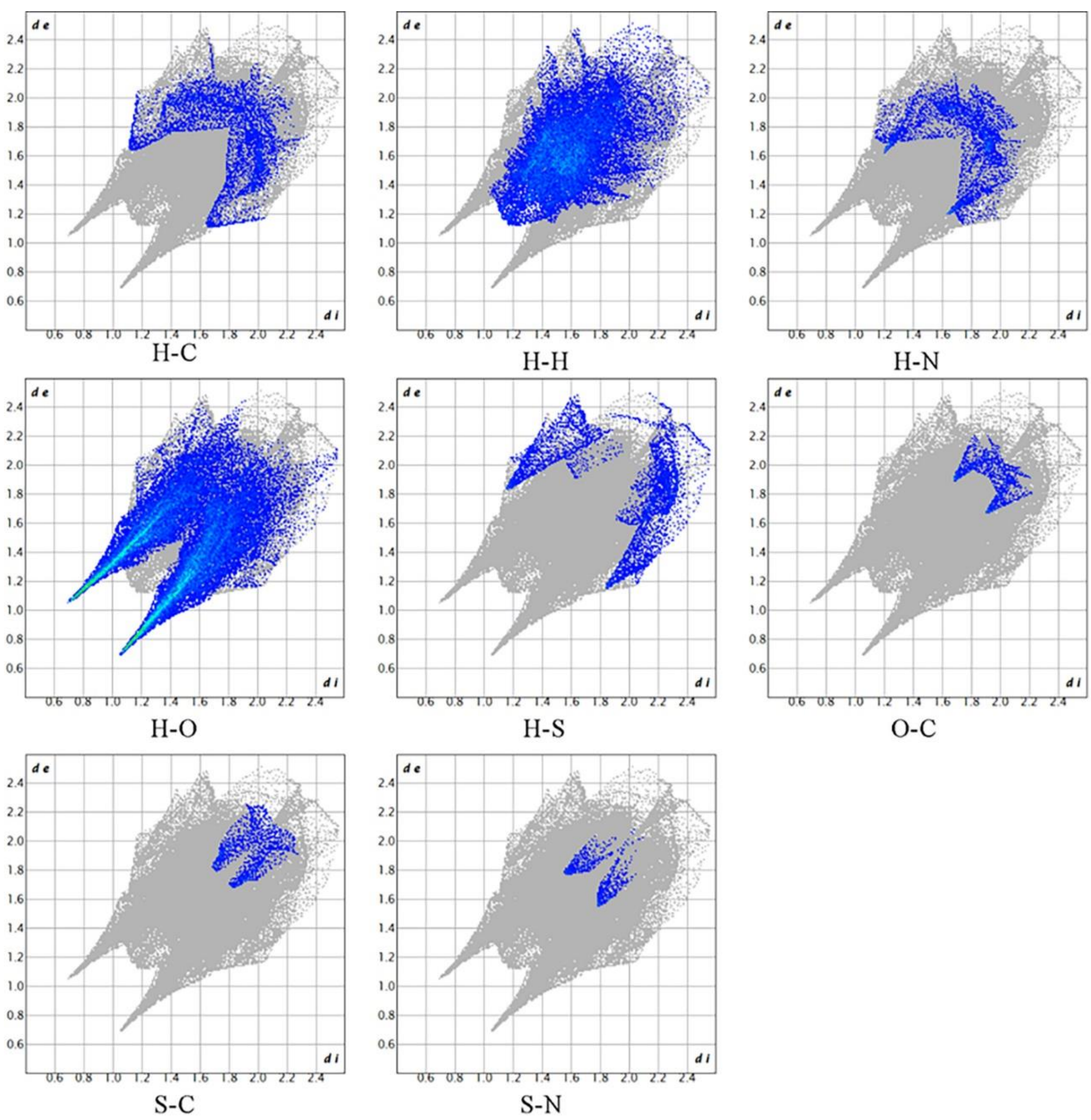

Figure S3. The fingerprint plots for the specific interactions within the Hirshfeld surface analysis of compound 2.

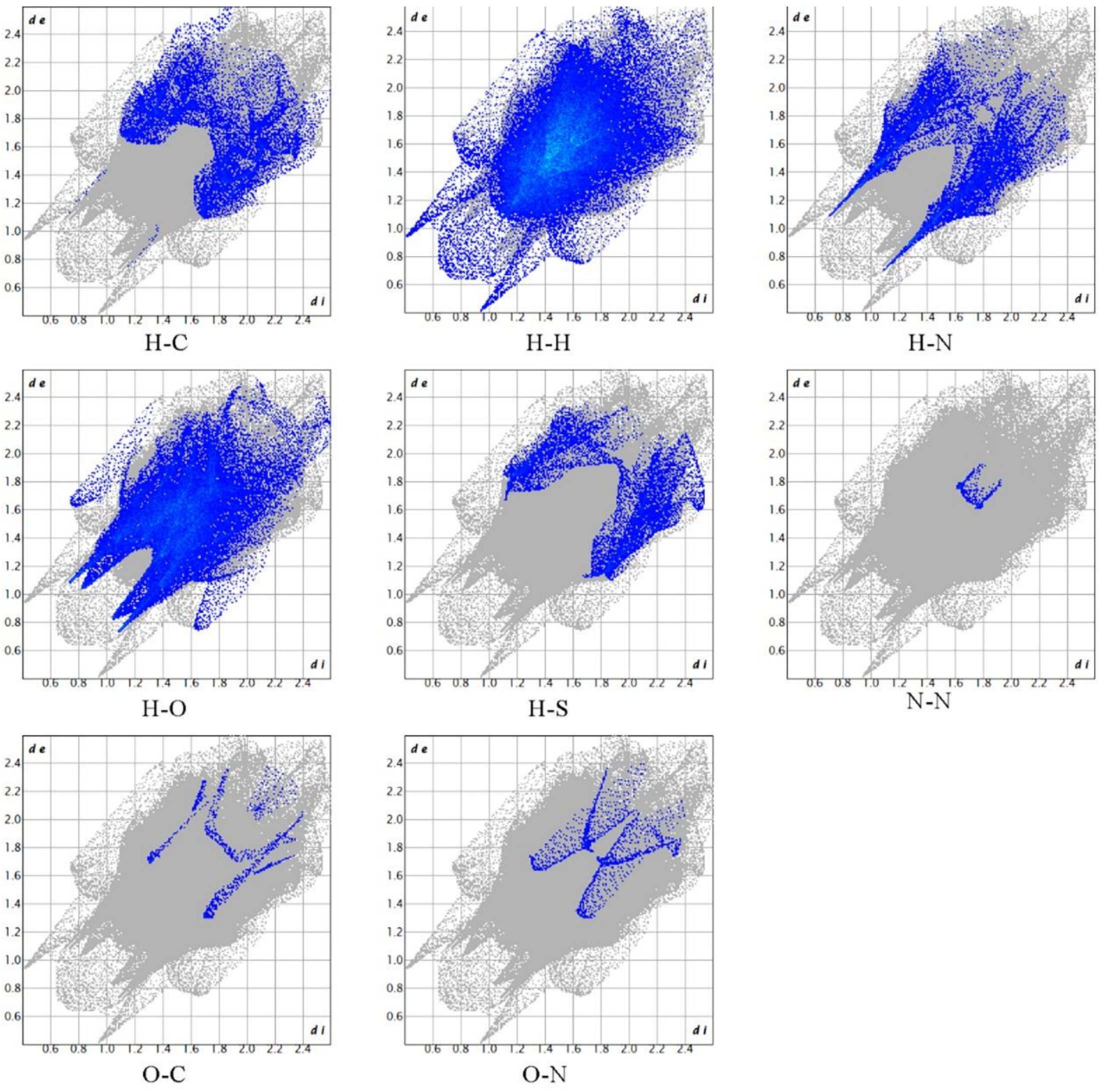

Figure S4. The fingerprint plots for the specific interactions within the Hirshfeld surface analysis of compound **1**.

Table S1. Crystallographic and optimized bond lengths (Å) for compound **2**.

Bond	Crystallographic	Optimized
Zn-O3	2.018	2.130
Zn-S2	2.320	2.442
Zn-O8	1.943	1.996
Zn-O9	2.066	2.129
Zn-N19	2.193	2.213

O8-C28	1.287	1.291
C28-C22	1.426	1.438
C22-C32	1.488	1.491
C22-N12	1.329	1.339
N12-C23	1.358	1.361
C23-C25	1.368	1.378
C25-C29	1.511	1.520
C26-O6	1.430	1.427
C25-C20	1.422	1.429
C20-C28	1.426	1.435
C20-C26	1.456	1.455
C26-N19	1.294	1.299
N19-N14	1.372	1.371
N14-C21	1.360	1.364
C21-N16	1.316	1.336
C21-S2	1.709	1.710

Table S2. Crystallographic and optimized bond angles (°) for compound **2**.

Angle	Crystallographic	Optimized
O3-Co-O8	106.3	107.3
O3-Co-O9	96.3	92.6
O3-Co-S2	104.3	95.9
O3-Co-N19	96.2	126.5
O9-Co-O8	89.8	92.4
O9-Co-S2	98.0	96.0
O9-Co-N19	167.0	126.3
O8-Co-S2	147.4	165.7
O8-Co-N19	83.2	84.1
S2-Co-N19	82.5	81.6
Co-O8-C28	132.5	134.9
O8-C28-C22	116.0	116.7
O8-C28-C20	126.3	125.5

C28-C22-C32	120.2	121.9
C28-C22-N12	120.0	118.4
C32-C22-N12	119.8	119.8
C22-N12-C23	123.6	125.2
N12-C23-C25	119.9	119.7
C23-C25-C29	117.6	117.3
C23-C25-C20	120.0	119.0
C25-C29-O6	108.7	109.3
C25-C20-C28	118.9	119.8
C25-C20-C26	119.4	117.8
C20-C26-N19	122.1	125.0
C26-N19-N14	116.7	116.5
N19-N14-C21	119.1	121.9
N14-C21-S2	123.4	123.7
N14-C21-N16	116.7	116.4
N16-C21-S2	119.9	120.0

Table S3. Crystallographic and optimized bond lengths (Å) for compound **1**.

Bond	Crystallographic	Optimized
Co1-O5	1.897	1.929
C1-N12	1.887	1.898
Co-N7	1.877	1.922
Co-N10	1.885	1.907
Co-O4	1.911	1.924
Co-N8	1.882	1.923
N7-N13	1.383	1.344
N13-C20	1.351	1.348
C20-N10	1.303	1.318
C20-S3	1.748	1.768
S3-C41	1.786	1.824

N7-C16	1.298	1.310
C16-C19	1.439	1.438
C19-C27	1.421	1.426
C27-C28	1.509	1.516
C28-O6	1.422	1.420
C27-C31	1.369	1.378
C31-N9	1.355	1.358
N9-C24	1.338	1.350
C24-C35	1.490	1.494
C24-C18	1.427	1.422
C18-O4	1.297	1.293
C49-S2	1.800	1.828
S2-C25	1.761	1.767
C25-N12	1.315	1.320
C25-N11	1.338	1.346
N11-N8	1.386	1.349
N8-C21	1.292	1.309
C21-C26	1.445	1.438
C26-C23	1.425	1.443
C23-O5	1.297	1.289
C23-C33	1.421	1.425
C33-C45	1.485	1.493
C33-N15	1.336	1.350
N15-C39	1.347	1.360
C39-C34	1.362	1.378
C324-C53	1.509	1.520
C53-O14	1.382	1.424
C34-C26	1.419	1.429

Table S4. Crystallographic and optimized bond angles (^{o}) for compound **1**.

Angle	Crystallographic	Optimized
O5-Co-N8	96.2	94.4

O5-Co-O4	87.5	88.4
O5-Co-N10	91.2	90.2
O5-Cp-N7	88.3	89.4
O5-Co-N12	177.7	175.8
N8-Co-N10	81.5	95.0
N8-Co-N7	174.0	174.9
N8-Co-N12	81.5	81.5
N8-Co-O8	88.6	89.1
N12-Cp-O4	93.0	90.5
N12-Co-N7	93.9	94.8
N12-Co-N10	88.4	91.2
O4-Co-N7	95.6	94.5
O4-Co-N10	177.6	175.7
N7-Co-N10	82.3	81.5
Co-O5-C23	123.1	125.7
O5-C23-C26	126.1	126.1
O5-C23-C33	115.8	115.8
C23-C33-N15	118.9	119.3
C23-C33-C45	121.4	121.2
C45-C33-N15	119.7	119.5
C33-N15-C39	124.2	124.1
N15-C39-C34	120.2	119.8
C39-C34-C26	119.4	119.9
C39-C34-C53	118.7	117.6
C34-C53-O14	112.5	113.8
C34-C26-C23	119.2	118.8
C34-C26-C21	118.1	118.7
C21-N8-N11	118.1	117.8
C21-N8-Co	125.7	126.5
Co-N8-N11	116.2	115.6
N8-N11-C25	107.9	110.0
N11-C25-N12	121.6	121.1

N11-C25-S2	121.1	118.9
C25-S2-C49	102.4	102.8
N12-C25-S2	117.3	120.0
Co-O4-C18	123.6	125.7
O4-C18-C19	125.6	126.0
C18-C24-C35	122.6	121.4
C18-C24-N9	119.0	119.0
C35-C24-N9	118.4	119.5
C24-N9-C31	124.3	124.6
N9-C31-C27	119.8	119.3
C31-C27-C28	119.7	118.3
C31-C27-C19	119.6	120.1
C27-C28-O6	109.5	108.8
C28-C27-C19	120.7	121.6
C27-C19-C18	119.3	118.9
C27-C19-C16	117.8	118.7
C19-C16-N7	123.9	124.9
C16-N7-N13	118.0	118.1
C16-N7-Co	126.4	126.3
Co-N7-N13	115.5	115.7
N7-N13-C20	108.4	110.2
N13-C20-N10	121.5	121.2
N13-C20-S3	111.7	112.5
C20-N10-Co	105.0	111.3
N10-C20-S2	126.9	126.3
C20-S3-C41	105.0	102.1

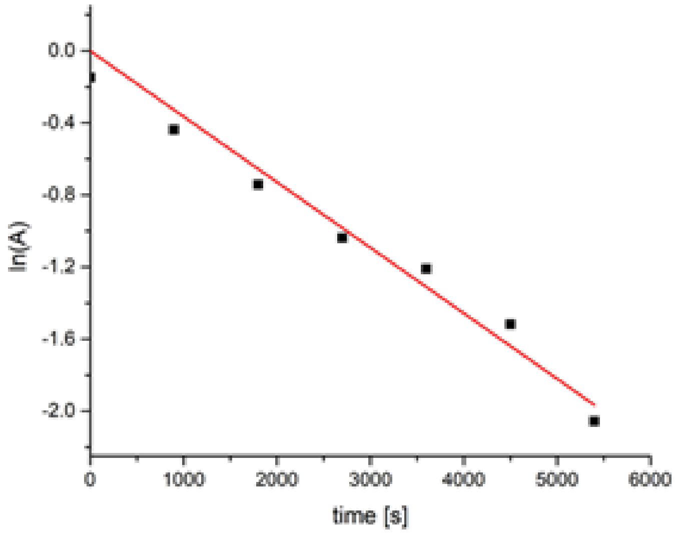

Figure S5. The kinetic curve for the reduction of methylene blue in presence of compound 1.

Table S5. Reduction wave potentials of [Co(PLITSC-H)$_2$]BrNO$_3$ ·CH$_3$OH and ([Zn (PLTSC)(H$_2$O)$_2$]SO$_4$·H$_2$O) versus Ag|AgCl[NBu4][BF4]–DMF.

**Complexes**	[Co(PLITSC-H)$_2$]BrNO$_3$ ·CH$_3$OH			[Zn(PLTSC)(H$_2$O)$_2$]SO$_4$·H$_2$O	
**Reduction peak**	Co(III)/Co(II)	Co(II)/Co(I)	Co(I)/Co(0)	Zn(II)/Zn(I)	Zn(I)/Zn(0))
**E /V Ag^+/AgCl**	-1.55	-1.15	-0.75	- 0.1	-1.3
